# Outcomes after HSCT for mucolipidosis II (I-cell disease) caused by novel compound heterozygous GNPTAB mutations

**DOI:** 10.3389/fped.2023.1199489

**Published:** 2023-07-06

**Authors:** Si-jia He, Dong-jun Li, Wen-qiong Lv, Wen-hao Tang, Shu-wen Sun, Yi-ping Zhu, Ying Liu, Jin Wu, Xiao-xi Lu

**Affiliations:** ^1^ Department of Pediatrics, West China Second University Hospital, Sichuan University, Chengdu, China; ^2^ Department of Pediatrics, Prenatal Diagnosis Center of West China Second University Hospital, Chengdu, China

**Keywords:** mucolipidosis type II (MLII), GNPTAB, sanger sequencing, hematopoietic stem cell transplantation, treatment

## Abstract

**Background:**

Mucolipidosis type II (MLII), or I-cell disease, is a rare lysosomal storage disease (LSD) caused by variants in the *GNPTAB* gene. MLII patients exhibit clinical phenotypes in the prenatal or neonatal stage, such as marked dysmorphic features, cardiac involvement, respiratory symptoms, dysostosis multiplex, severe growth abnormalities, and mental and motor developmental abnormalities. The median age at diagnosis for MLII is 0.7 years, the median survival is 5.0 years, and the median age at death is 1.8 years. No cure for MLII exists.

**Methods:**

Sanger sequencing of the *GNPTAB* gene identified the compound heterozygous mutations c.673C > T in exon 7 and c.1090C > T in exon 9, which were novel double heterozygous mutations first reported in China. For the first time, we describe our experience in the use of HSCT for MLII. Our patient underwent HSCT with cells from a 9/10 human leukocyte antigen (HLA)-matched unrelated donor at 12 months of age. Myeloid neutrophil and platelet engraftment occurred on Days 10 and 11, respectively.

**Results:**

The patient's limb muscle tension was significantly reduced, and his gross and fine motor skills were improved four months after transplantation. DST(Developmental Screen Test) results showed that the patient's fine motor skills and mental development were improved compared with before HSCT.

**Conclusion:**

MLII is a very severe lysosomal storage disease, to date, only 3 cases have been reported on the use of HSCT to treat MLII. Our data show that HSCT is a potential way to prolong the life of patients and improve their quality of life. Due to the lack of comparable data and time, the exact benefit remains unclear in MLII patients. Longer-term follow-up and in-depth prospective studies are indispensable.

## Introduction

Mucolipidosis type II (MLII), or I-cell disease, is a lysosomal storage disease (LSD) caused by variants in the *GNPTAB* gene on chromosome 12q23.2 and generally results in death before the age of 10 years (1, 2). The *GNPTAB* gene encodes the alpha and beta subunits of N acetylglucosamine-1 (GlcNAc) phosphotransferase (EC 2.7.8.17) localized in the Golgi apparatus, which is essential for normal processing and packaging of soluble lysosomal enzymes by initiating the first step of tagging lysosomal enzymes with mannose-6-phosphate (M6P) (3, 4). There are four types of ML, which mostly are classified according to the enzymes that are deficient or mutated and MLII is the most severe form of ML and results from a complete loss of GlcNAc phosphotransferase activity (5, 6). MLIV is caused by mutations in *MCOLN1*, that encodes mucolipin-1 and is belonging to the transient receptor potential (TRP) gene family (7). MLII patients exhibit clinical phenotypes in the prenatal or neonatal stage, such as marked dysmorphic features, cardiac involvement, respiratory symptoms, dysostosis multiplex, severe growth abnormalities, and mental and motor developmental abnormalities. The median age at diagnosis for MLII is 0.7 years, the median survival is 5.0 year, and the median age at death is 1.8 years (8). Death often occurs in the first decade of life from cardiopulmonary disease (9).

The clinical diagnosis of MLII can be confirmed by biochemical analysis of patient´s material. Because arylsulfatase A and *β*-glucuronidase are transported to lysosomes in an M6P-dependent pathway, biochemical criteria including an at least tenfold increase in plasma lysosomal enzyme levels (arylsulfatase A and *β*-glucuronidase activities) could be used as a reference index for the diagnosis of ML (10). Targeted sequencing of *GNPTAB* is finally required to confirm the clinical diagnosis of MLII.

There are currently no curative or disease-modifying treatments available for MLII, not even in the context of clinical trials. With the rapid development of hematopoietic stem cell transplantation (HSCT), it has been performed for over 30 years in the treatment of various lysosomal storage disorders. Evidence on the efficacy of HSCT in MLII is still scarce. The combined international experience in the use of HSCT for MLII are only 23 patients, and only 3 cases have been reported in detail (11–13). Hematopoietic donor cells can produce functional M6P-containing lysosomal enzymes, which can be taken up intracellularly and transported to lysosomes for the degradation of macromolecules (14).

This report summarizes the case of a 16-month-old ML patient with novel double heterozygous mutations in the GNPTAB gene who underwent HSCT at 12 months of age in China.

## Materials and methods

### Patient and diagnosis

**Figure 1:**
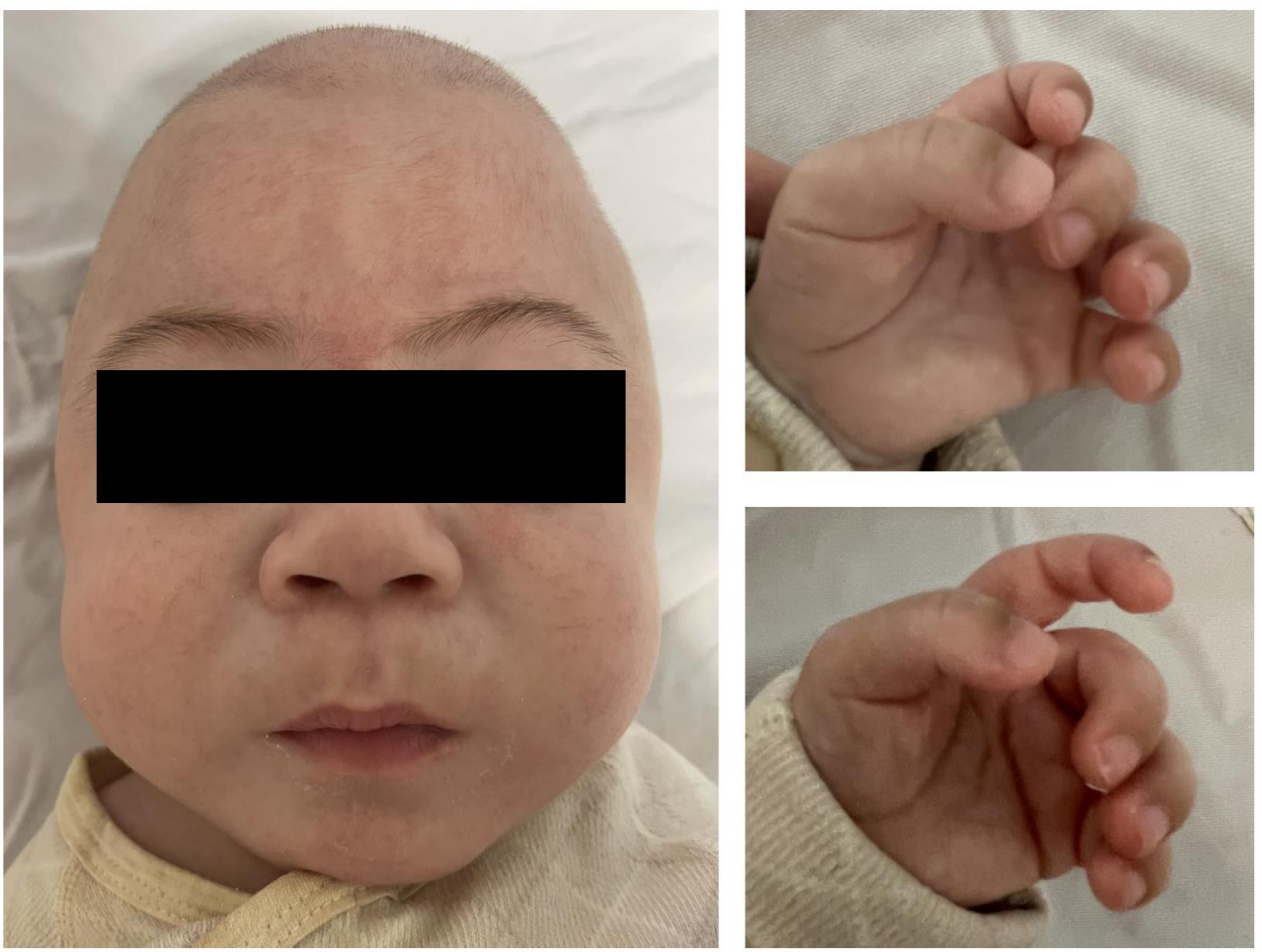
Abnormal facial features (puffy eyelids, epicanthus, flat nasan bridge, anteverted nostrils, gingival hyperplasia and macroglossia).

**Figure 2:**
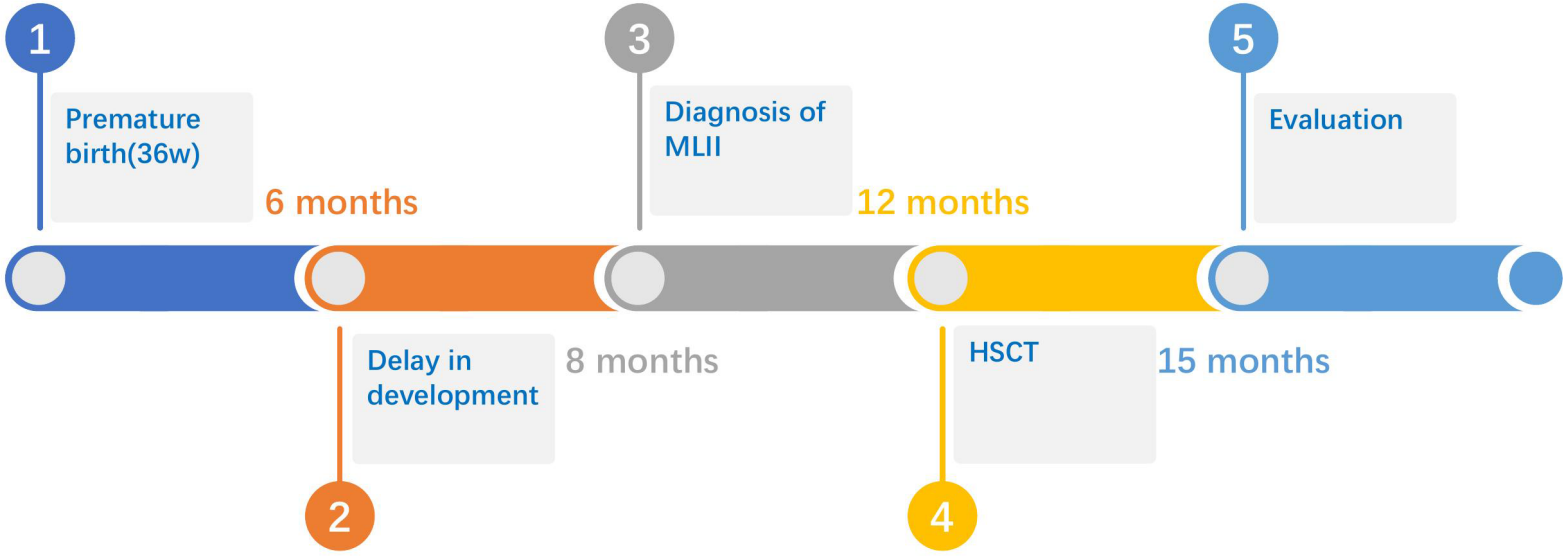
The time check point of the patient history.

The male patient is the first child of nonconsanguineous healthy parents. He was born premature (36 weeks) ﻿and was delivered by cesarean section because of little amniotic fluid. The newborn presented with a birth weight of 2,800 g (50th percentile), a body length of 48 cm (50th percentile) and an occipitofrontal circumference (OFC) of 32 cm (25th percentile). The postnatal physical examination confirmed thoracic deformity, a large inguinal hernia, a prominent forehead, puffy eyelids, epicanthus, a flat nasal bridge, anteverted nostrils, gingival hyperplasia, macroglossia and contractures of all large joints (Figure 1). Neonatal screening test results, including 17*α*-hydroxyprogesterone and thyroid-stimulating hormone (TSH), were all normal. This patient first visited our hospital when he was 6 months old, presenting with unstable head erection, an inability to support the weight of his lower limbs, and decreased muscle tension and reflexes of the limbs. At the age of 8 months, his parents found that his motor development was significantly behind that of children of the same sex and age, and he was still unable to raise his head, turn over or sit (Figure 2). In addition, he showed worsened limb joint contractures, decreased bilateral limb muscle tension, and fair and rough skin. Hepatosplenomegaly or other obvious abnormalities were not observed in this patient.

**Figure 3:**
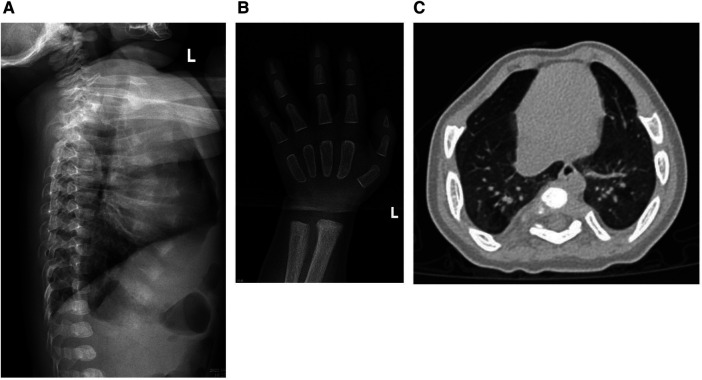
(**A**) The x-ray shows the spine have kyphosis at the junction of the thoracolumbar segment. Thoracic and lumbar physiological curvature developmental abnormalities. (**B**) The cartilage development of wrist joint was abnormal. (**C**) The chest was deformed and the interstitial changes of the lungs were obvious.

**Figure 4:**
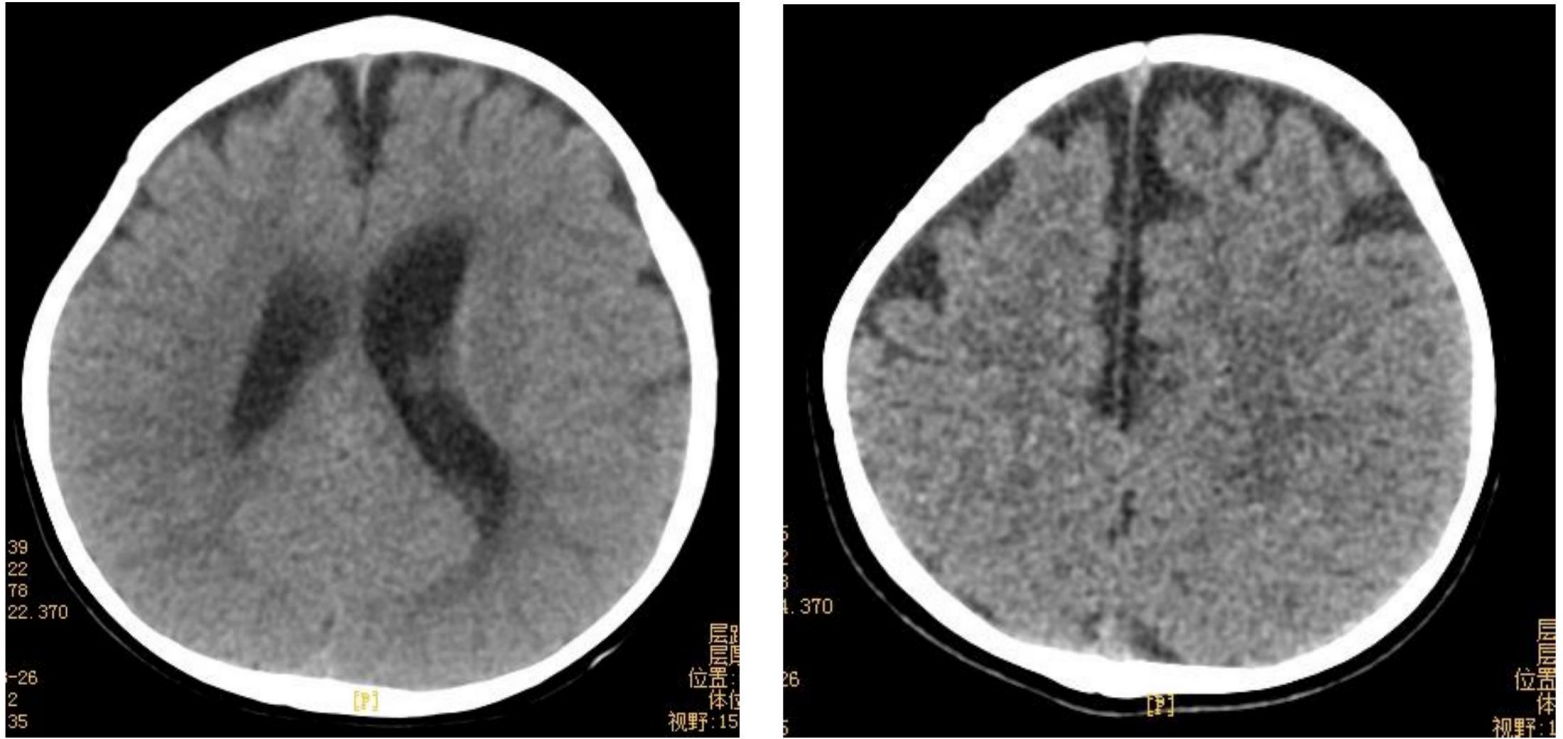
Cranial CT showed abnormal skull development and bilateral ventriculomegaly. Bilateral frontotemporal extracerebral Spaces were widened.

**Figure 5:**
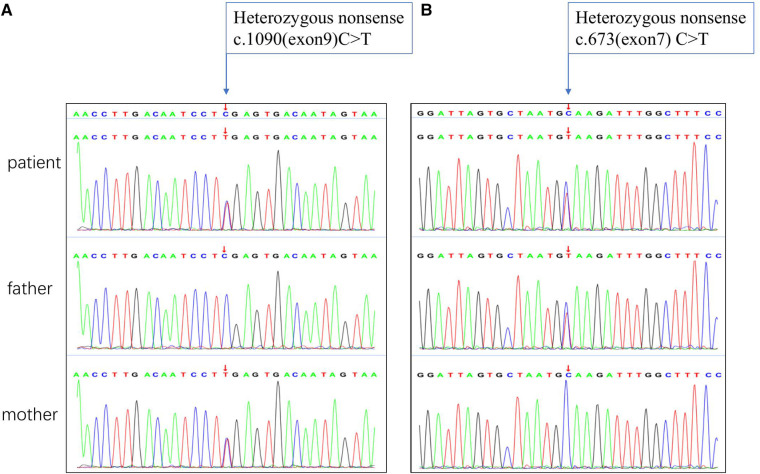
Sanger sequencing chromatograms of the *GNPTAB* gene variants in this patient. (**A**) Homozygous nonsense of c. 1090C > T mutation marked by arrow inherited from the mother. (**B**) Homozygous nonsense of c.673C > T mutation marked by arrow inherited from the father.

The results of biochemical tests and routine blood, bowel and urine tests were all negative. Characteristic features of skeletal dysplasia were observed in vertebral side x-ray imaging (Figure 3). A cranial CT scan showed ventriculomegaly and abnormal skull development (Figure 4). Echocardiography revealed that the foramen ovale was patent, and the EF was normal. The development screening test (DST) showed that the patient's mental and motor development were significantly delayed (Table 1). Standard biochemical testing of lysosomal enzyme activities, including arylsulfatase A and *β*-glucuronidase, in this patient's serum identified a more than tenfold increase (Table 2). Whole Exome sequencing was performed using IDT The xGen Exome Research Panel v2.0. The genetic data were analyzed and screened by the cloud platform of genetic disease precision diagnosis integrating molecular biology annotation, biology, genetics and clinical characteristics analysis, and the pathogenicity was evaluated by the American College of Medical Genetics (ACMG) gene variant classification system. Finally, Sanger sequencing was used to verify the pathogenic mutation. Sanger sequencing of the *GNPTAB* gene identified the compound heterozygous mutations c.673C > T in exon 7 and c.1090C > T in exon 9, both pathogenic according to the American College of Medical Genetics and Genomics (ACMG) criterion, and were novel double heterozygous mutations first reported in China (Table 3) (Figure 5). The loss-of-function mutations in both alleles predicted the manifestation of a severe phenotype of MLII.

**Table 1:** The Development screen test (DST) in the patient before and after transplantation.

Development Screen Test for ages 0–6						
Months of ages	Movement	5	Social adaptation	4	Intelligence	10
6	Mental index original points	11	Mental index	60	Age mental	4
	Developmental quotient original points	15	Developmental quotient	<50	Development of ages	3
15	Movement	8	Social adaptation	8	Intelligence	21
	Mental index points	21	Mental index	<50	Age mental	8
	Developmental quotient original points	37	Developmental quotient	48	Development of ages	7

**Table 2:** Standard biochemical testing of lysosomal enzyme activities including arylsulfatase A and *β*-glucuronidase in this patient’s serum.

Name of enzyme	Patient (10 month)	Patient (15 month)	Mother	Father	Normal control
arylsulfatase A (nomol/17 h/*μ*l)	147.38	129.17	11.19	13.53	10.8
*β*-glucuronidase (nomol/17 h/*μ*l)	72.79	77.12	11.45	8.82	3.98

**Table 3:** Sanger sequencing of the *GNPTAB* gene identified the compound heterozygous mutations c.673 > T in exon and c.1090 > T in exon 9 in this patient, and all mutations were pathogenic.

Gene	Type of mutation	Nucleotide change	Amino acid change	Exons	OMIM clinical phenotype	American College of Medical Genetics classification
GNPTAB	Nonsense	763C > T	NM_024312: p.Q225*,1032(p.Gln225Ter,1032)	Exon 6	Mucolipidosis II alpha/beta/AR; Mucolipidosis III alpha/beta	PVS1 + PM2 + PM3
GNPTAB	Nonsense	1090C > T	NM_024312: p.R364*,893(p.Arg364Ter,893)	Exon 9	Mucolipidosis II alpha/beta/AR; Mucolipidosis III alpha/beta	PVS1 + PM2 + PM3

### Hematopoietic stem cell transplantation (HSCT)

Considering the young age at diagnosis in this patient and the parents' strong desire, HSCT was the only choice to be made after parents were well informed of the limitations and potential risks. Written informed consent was obtained from both parents. This patient underwent HSCT at 12 months of age. He received unrelated umbilical cord blood from Zhejiang Cord Blood Bank, which was 9/10 human leukocyte antigen (HLA) matched. The donor had the same blood type as the recipient (O Rh pos). The nucleated and CD34 cell doses of the infused CB were 1.697 × 10^8^/kg and 3.68 × 10^6^/kg, respectively. The CBT preparative regimen consisted of ATG (rabbit anti-human thymocyte immunoglobulin) 2.5 mg/kg/d (Days -10, -9), fludarabine 30 mg/m^2^/day (day -9, -8, -7, -6, -5), cyclophosphamide 50 mg/kg/day (day -3, -2), Busulfex 4.0 mg/kg/d (day -8, -7, -6, -5). For graft-vs.-host disease (GVHD) prophylaxis, tacrolimus, and methylprednisolone were administered.

## Results

### Genetic analysis

Sanger sequencing was found two heterozygous variants (c.673C > T in exon 7 and c.1090C > T in exon 9) in this patient. Heterozygous *GNPTAB* (NM_024312) c.637C > T (p.Q225*,1,032) and c.1090C > T (p.R364*,893) variants were detected in the patient. The c.637C > T (p.Q225*,1,032) variant was detected in heterozygous state in the father, was a novel nonsense variant (Table 3). The other variant c.1090C > T (p.R364*,893) has been previously reported to appear frequent among populations in China, and lead to a premature stop codon, which may activate nonsense-mediated mRNA decay (15). The bioinformatic prediction of pathogenicity and the analysis of inheritance patterns and OMIM clinical phenotypes all suggested that these mutations caused the disease. Variants c.673C > T and c.1090C > T were both pathogenic (PVS1 + PM2 + PM3) according to the American College of Medical Genetics and Genomics (ACMG) criteria.

### HSCT-related complications and engraftment

The pretransplantation myeloablative conditioning regimen was well tolerated by the patient. Because of the young age, this patient still developed acute GvHD of the skin (grade III), which was steroid sensitive and resolved. Myeloid neutrophil and platelet engraftment occurred on Days 10 and 11, respectively. The engraftment remained stable with complete donor chimerism.

### Manifestations of MLII despite HSCT and activity reduction of lysosomal enzymes including arylsulfatase A and *β*-glucuronidase

When this patient was 6 months old, he was still unable to erect his neck, and the muscle tone of his limbs was reduced, and he could not turn over and support himself. The development screening test (DST) showed the development of ages around 3 months old. At the age of 8 months, the patient's gross motor development was significantly delayed, and he could not turn over, crawl, or sit alone. At the same time, fine motor development and mental retardation were manifested as the inability to change small objects alone, no obvious response to names, and unable to laugh. The patient's overall development progressed continuously after HSCT (Figure 2). At the age of 14 mouths, 2 mouths after HSCT, the parents felt that the skin of the patient was more delicate than before. Even though he was able to raise his head slightly, the muscle tension of the limbs was significantly lower than before, the joint contracture caused by continuous muscle contraction was better, and the muscle state was more relaxed, therefore, the range of motion of the limbs was increased than before. In general, the parents felt that the patient's muscle strength and joint range of motion were significantly improved. Fine motor skills were poorly adapted, and more practice in daily life is needed. Nevertheless, the development screening test (DST) showed significant progression when the patient was 15 months old (Table 1). As we expected, the relevant HSCT-mediated effect was partly achieved concerning lysosomal enzyme activities because lysosomal enzymes secreted by the donor cells were taken up by defective MLII cells. The activities of lysosomal enzymes, including arylsulfatase A, were partly reduced 3 months after HSCT. Because of the disruption of the pathway by which the M6P signal is associated with numerous enzymes, *β*-glucuronidase was slightly changed from before (Table 2).

## Discussion

Mucolipidosis II alpha/beta is caused by variants in the *GNPTAB* gene, which encodes the *α*/*β*-precursor of GlcNAc-1-phosphotransferase. The clinical manifestations of patients with MLII alpha/beta are similar to those observed in some LSDs, such as MPS, which could show physical characteristics, such as coarse facial features, short stature, skeletal deformity, hepatosplenomegaly, and a reduced life expectancy (9). In MLII alpha/beta, frameshift or nonsense variants are often the most frequent mutation type and cause a complete loss of enzyme activity, leading to a more severe clinical phenotype (1, 16). Cathey, Leroy (10) previously reported that p. R364X (c.1090C > T) in exon 9 was the most frequent mutation in both MLII *α*/*β* and MLIII *α*/*β* patients, and homozygous patients could have a clinically severe phenotype of ML. GNPTAB mutations have been reported to cluster regionally, and patients carrying the variant c.1090C > T most were from China (15).

In the present manuscript, we report the case of a patient who has compound heterozygous pathogenic variants of the *GNPTAB* gene. The c.673C > T variant in exon 7 was inherited from the father, and the c.1090C > T variant in exon 9 was inherited from the mother. It is worth mentioning that one of the mutation types identified in this patient has not been reported thus far. According to ACMG/AMP criteria, this nonsense mutation was classified as a pathogenic variant (PVS1 + PM2 + PM3). Kudo, Brem (17) previously reported that MLII *α*/*β* is defined by two severe mutations, and Qian, van Meel (18) presented that the stealth domain mediates the catalytic function of GlcNAc-1-phosphotransferase. Thus, in our study, the patient with two nonsense mutations had the clinical characteristics of MLII *α*/*β*, with early onset at 8 months old, and these mutations may have caused his severe enzyme deficiency.

Since there is no effective treatment for MLII, the recognition of typical clinical characteristics is very important for prenatal diagnosis and the prevention of this disease.

Symptomatic treatment is applicable to almost all LSDs, and the main treatment method includes surgical treatment for bone deformity and hernia and﻿ heart valve replacement (15, 19). Hematopoietic stem cell transplantation (HSCT) has been used for the metabolic “cross-correction” of lysosomal storage diseases for over 30 years, and it has been performed in a few MLII patients, although comprehensive follow-up data are extremely limited (20, 21). Previous reports indicated the combined international experience in the use of HSCT for MLII have 23 patients, and the overall survival was still low (26%). There only 3 cases have been reported on the use of HSCT to treat MLII (11, 22). HSCT might have had an impact on gross motor function recovery, neurocognitive function remodeling and quality of life improvement in this patient. This is the first report to specify the short-term effects of experimental HSCT in an MLII patient in China. HSCT aims to provide donor-derived hematopoietic cells to produce functional M6P-containing lysosomal enzymes in patients with lysosomal storage disorders, allowing for intracellular uptake with appropriate trafficking to the lysosome for substrate degradation (23, 24). Because of the amount of tissue damaged by the time of transplantation, the age for HSCT is also considered to be a crucial predictor for success in progressive disorders (12). In mucopolysaccharidosis, patients undergoing HSCT earlier in life have been shown to have better cognitive outcomes and performance (25).

Due to the lack of a control group due to very limited natural history data in MLII, we faced several challenges when evaluating the therapeutic effect of HSCT in this patient. Lund, Cathey (22) performed a retrospective study on 22 transplanted MLII patients and indicated that the overall survival at the last follow-up was only 27%, with a median time to death of 27.6 months. Three of these patients received bone marrow from siblings, and two underwent haploidentical HSCT with a parent as a donor. Most patients still die due to organ failure later after transplantation because the impact of HSCT on orthopedic and cardiac valve pathology may be limited (26). Airway pathology in MLII is possibly related to the altered differentiation of tracheal cartilage (27). The most frequent reported causes of death in MLII patients still pulmonary and or cardiac complications to multiorgan failure, and a few cases of death were complications after stem cell transplantation (8). Shibazak et al. (28) reported that published case reports indicate the preservation of cognitive function in follow-ups after transplantation.

In our patient, one month after HSCT, the patient's skin roughness was significantly improved. At the same time, four months after transplantation, the patient's limb muscle tension was significantly reduced, and the gross and fine motor skills were improved. The patient was tested with the DST (development screening test) at chronological ages of 6 and 15 months. The DST results showed that the patient's fine motor skills and mental development were improved compared with before HSCT. The functional M6P-containing lysosomal enzymes which was produced by hematopoietic donor cells, are transported to the lysosomes for degradation of macromolecule. Thus, metabolic “cross-correction” is achieved. In MLII patient, numerous enzymes which are associated with M6P signal are disrupted. This results in inappropriate secretion and insufficient delivery of multiple enzymes to the lysosome. Due to the inappropriate plasma secretion of several enzymes in MLII patients, the evaluation of lysosomal enzyme activity may remain significantly elevated months to years after successful HSCT (22). This may explain the fact that not all enzyme activities were reduced after HSCT in our patient (Table 2). Dogterom et al. (8) indicated that as MLII is an extremely rare disease, it could be analyzed only based on recent case reports, and they concluded that the median age at death was 1.8 years for the MLII phenotype. Although the most frequent causes of death in MLII are pulmonary and/or cardiac complications, HSCT is still a potential way to impact cellular function and aim to appropriately prolong the life of patients and improve their quality of life (14). Therefore, longer-term follow-up of this patient is warranted, combined with reassessments of motor and mental development.

## Data Availability

The data analyzed in this study was obtained from a third-party inspection platform, the following licenses/restrictions apply: protection of intellectual property rights of the original data by the gene company. Requests to access these datasets should be directed to Xiaoxi Lu, lu_helena@sina.com.
